# Experiences of sexual well-being interventions in males affected by genitourinary cancers and their partners: an integrative systematic review

**DOI:** 10.1007/s00520-023-07712-8

**Published:** 2023-04-14

**Authors:** Kathryn Schubach, Theo Niyonsenga, Murray Turner, Catherine Paterson

**Affiliations:** 1 Faculty of Health, University of Canberra, Bruce ACT, Australia; 2 Rehabilitation, Activity, Cancer, Exercise and Survivorship (PACES) Research Group, University of Canberra, Bruce ACT, Australia; 3 Robert Gordon University, Aberdeen, Scotland, UK

**Keywords:** Sexual well-being, Genitourinary cancers, Intervention, Lived experience, Systematic review

## Abstract

**Purpose:**

Sexual well-being has been identified as an unmet supportive care need among many individuals with genitourinary (GU) cancers. Little is known about the experiences of using sexual well-being interventions among men and their partners.

**Methods:**

This review was reported using the Preferred Reporting Items for Systematic Reviews and Meta-Analyses (PRISMA) and followed a systematic review protocol. Data extraction and methodological quality appraisal were performed, and a narrative synthesis was conducted.

**Results:**

A total of 21 publications (reporting on 18 studies) were included: six randomised control trials, seven cross-sectional studies, three qualitative studies, and five mixed methods studies. Sexual well-being interventions comprised medical/pharmacological and psychological support, including counselling and group discussion facilitation. The interventions were delivered using various modes: face-to-face, web-based/online, or telephone. Several themes emerged and included broadly: (1) communication with patient/partner and healthcare professionals, (2) educational and informational needs, and (3) timing and/or delivery of the interventions.

**Conclusion:**

Sexual well-being concerns for men and their partners were evident from diagnosis and into survivorship. Participants benefited from interventions but many articulated difficulties with initiating the topic due to embarrassment and limited access to interventions in cancer services. Noteworthy, all studies were only representative of men diagnosed with prostate cancer, underscoring a significant gap in other GU cancer patient groups where sexual dysfunction is a prominent consequence of treatment.

**Implications for cancer survivors:**

This systematic review provides valuable new insights to inform future models of sexual well-being recovery interventions for patients and partners with prostate cancer, but further research is urgently needed in other GU cancer populations.

**Supplementary Information:**

The online version contains supplementary material available at 10.1007/s00520-023-07712-8.

## Introduction

Genitourinary (GU) cancers are located within the urinary and reproductive systems. The incidence rate of detection is 37.5 per 100,000 individuals affected by prostate cancer, bladder cancer is 11.7, and kidney 7.8, and both penile and testicular are less common at approximately 2 per 100,000 men [1]. Improvements in diagnostic tests and treatment options for GU cancers have improved survival rates [2–5]. However, all treatment modalities for GU cancers can negatively impact sexual function at some stage in the cancer trajectory, given the invasive nature of treatments [1, 6, 7]. Existing systematic reviews among GU cancer populations [8–13] have all identified that patients affected by GU cancer continue to report unmet sexual well-being needs with a lack of support from healthcare professionals.

Despite the well-documented unmet sexual well-being needs in GU patient groups, various interventions are available in cancer services to treat sexual dysfunction. Such interventions include (1) pharmacological treatments such as phosphodiesterase-5 (PDE5) inhibitors, (2) mechanical devices such as vacuum pumps and penile implants, (3) psycho-educational interventions such as couples’ counselling, and (4) education and peer support [14–16]. Many individuals affected by GU cancers continue to experience sexual health concerns that negatively impact their physical, social, spiritual, and psychological well-being. When sexual health concerns and needs are not met in routine clinical services, it can lead to a reduction in the patients’ sexual motivation, intimacy, and self-esteem, resulting in partner distress, reduced relationship satisfaction, and a breakdown in communication between the couples [9–11, 17, 18].

Several barriers to engaging in sexual well-being interventions and recovery have been identified. Known barriers include (1) reluctance to initiate a conversation with their healthcare professional [19], (2) healthcare professionals report a lack of time to discuss sexual well-being during consultations [20, 21], (3) patients have expressed that if a clinician does not raise the topic during the consult, then it must not be a valid clinical concern [22], and (4) sexual dysfunction is an irreversible result of cancer treatments [14, 23]. Acknowledging these barriers, it is important to understand the experiences of available sexual well-being interventions embedded in a biopsychosocial framework [24, 25]. The biopsychosocial framework is important because it provides a holistic approach to managing sexual well-being and addressing what matters most to patients and their partners [24, 26].

It is imperative to focus on the patient’s perspective when developing and evaluating sexual well-being interventions. Patient-reported measures (PROMs) are tools utilised in clinical practice and research to gain insights from the patient’s perspective [27]. Self-reported measures and qualitative experiences can contribute to understanding the patients’ experiences and expectations of sexual well-being interventions and provide important insights into contemporary barriers and facilitators in different healthcare contexts to addressing sexual well-being concerns [28, 29].

This integrative systematic review aimed to understand the experience of sexual well-being interventions in people and their partners affected by GU cancer. Specifically, this review addressed the following clinically focussed research question:

In patients diagnosed with GU cancers, and their partners, what are their experiences of sexual well-being interventions?

## Methods

### Design

This integrative systematic review has been reported according to the Preferred Reporting Items for Systematic Reviews and Meta-Analyses (PRISMA) guidelines [30]. This review followed a systematic review protocol available upon request.

### Search strategy and pre-determined eligibility criteria

The following electronic databases (APA PsycINFO, CINAHL, Cochrane Library (Database of Systematic Reviews and CENTRAL Register of Controlled Trials), MEDLINE, and Scopus) were searched in November 2021. Limiters were applied to the search for date range 1997 onwards and for studies published in English (see Supplementary Table 1 for full record of database searches). Articles were included if they met the following pre-screening eligibility criteria.

### Eligibility criteria

#### Types of studies

##### Inclusion

Qualitative, quantitative, and mixed methods studies irrespective of research design.

##### Exclusion

Commentaries, editorials, non-peer-reviewed literature, systematic reviews, and non-English studies.

#### Types of participants

##### Inclusion

Adults > 18 years diagnosed with GU cancer (and partners) irrespective of time since diagnosis or treatment modality.

##### Exclusion

Studies conducted with participants with non-GU cancers.

#### Types of interventions

The interventions included (1) pharmacological therapy such as phosphodiesterase-5 (PDE5) inhibitors and intracavernosal injections—alprostadil, phentolamine, papaverine, intraurethral muse; (2) mechanical devices such as vacuum erectile devices and penile implants; and (3) psychosocial interventions including counselling, and couples counselling, mindfulness, and group therapy.

#### Types of outcome measures

The primary outcome was the experience of sexual well-being interventions as reported by patients and their partners.

#### Screening process

All articles identified were imported into Endnote referencing software and exported to Covidence Systematic Review software (Covidence© 2020, version 1517, Melbourne, Australia) for the removal of duplicates and to manage the article screening process. Reviewers applied a pre-eligibility criterion to all titles and abstracts, and any conflicts were resolved by discussion. Full-text articles were reviewed by authors and any disagreements resolved by discussion.

#### Data extraction

Data were extracted by one review author, and quality was checked by a second reviewer. A data extraction table was developed and piloted on a small number of studies first. The data extraction table contained information in relation to the participants’ clinical and demographic characteristics, setting, sample size, study design, data collection tools, and type of intervention. A second data extraction table was used for the qualitative data (see Supplementary Table 2).

#### Quality assessment

The methodological quality and evaluation of the studies were assessed using the Mixed Methods Appraisal Tool (MMAT) [31]. The Mixed Methods Appraisal Tool comprises 25 criteria and two screening questions, and any disagreements in assessment scores were resolved by discussion among the reviewers. This assessment tool was used because it enabled a plethora of research designs to be evaluated.

#### Data synthesis

This integrative review used the Whittemore and Knafl (2005) methodological approach to evidence synthesis. The data synthesis used an inductive analysis examining the collected data for patterns, similarities, and differences across the included studies [32]. Inductive analysis involved a process of data comparison and drawing and verifying relevant themes from primary sources [33]. The data reduction then was compiled into groups of sexual well-being interventions and data collection tools that evaluated patient experience. Next, the qualitative and quantitative data were synthesised to compare the similarities and differences [33]. The development of conclusions involved judgement decisions of the themes with verification using primary data for accuracy and validation.

## Findings

**Fig. 1:**
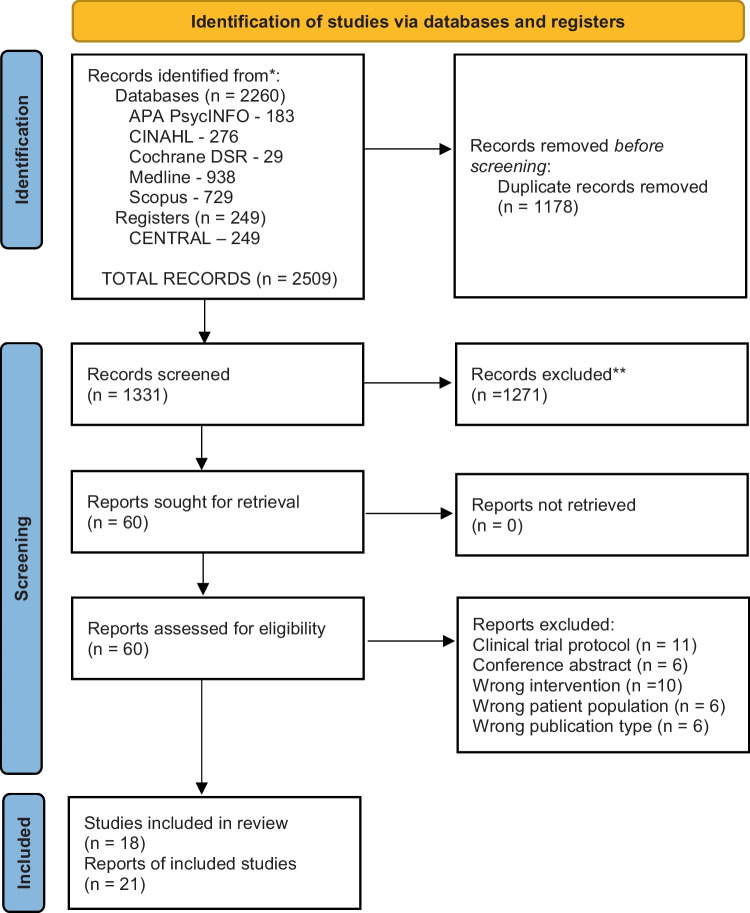
PRISMA 2020 flow diagram for new systematic reviews, which included searches of databases and registers only. From: Page MJ, McKenzie JE, Bossuyt PM, Boutron I, Hoffmann TC, Mulrow CD, et al. The PRISMA 2020 statement: an updated guideline for reporting systematic reviews. *BMJ* 2021;372:n71. https://doi.org/10.1136/bmj.n71; for more information, visit: http://www.prisma-statement.org/

There were 1131 articles screened, and 21 articles were included in the study (see Fig. 1). Of note, three articles reported on the same study [15, 34, 35], resulting in a total of 18 studies (see Fig. 1). The studies were conducted in a range of countries, which included Australia (*n* = 3), Brazil (*n* = 1), Canada (*n* = 3), Denmark (*n* = 1), France (*n* = 1), Netherlands (*n* = 1), the USA (*n* = 7), and UK (*n* = 1). The sample sizes ranged from 6 to 896, with a total sample of 2247 participants included. The participants’ mean age ranged from 60 to 67 years, and the partners’ mean age ranged from 57 to 65 years across the studies. Most participants had completed, at minimum, some form of high school education (Table 1). Noteworthy, all of the included studies were representative only of men with prostate cancer and lacked insights into the sexual well-being intervention experiences among other GU cancer populations.

**Table 1:** Overview of included studies

Author and year	Purpose	Setting	Sample size	Participants	Sampling	Response rate	Design	Time points	Data collection tools	Intervention
Bossio et al. 2021 Canada	To examine the feasibility of a mindfulness group aimed at improving sexual intimacy for couples following prostate cancer treatments	Prostate cancer survivorship program Vancouver General Hospital	*N* = 14 couples	Clinical: Couples with sexual dysfunction secondary to prostate cancer treatments In a relationship longer than × 1 year. Partners could be of any gender Demographics: Men’s mean age: 65.6 (52–75) All partners identified as women mean age of 61.4 (44–74) years Education: Some college/undergraduate degrees 28.6%	Convenience	Not reported	Mixed methods	3	Questionnaires: Demographics: unstructured questionnaire Relationship: Adapted dyadic adjustment scale (ADAS) Sexual functioning: Global measure of sexual satisfaction (GMSEX). International Index of Erectile Dysfunction (IIEF). Female Sexual Function Index (FSFI). Depression and anxiety Hospital Anxiety and depression scale (HADS) Five Facet Mindfulness Questionnaire short form (FFMQ-SF)	4 session mindfulness-based therapy group Principles of psychoeducation, mindfulness skills practice, cognitive behavioural therapy Groups were 2 h in length weekly Invited to complete homework 10–60 min each day
Chambers et al. 2014 Australia	To investigate the efficacy of couples-based peer-delivered telephone support or couples-based nurse-delivered telephone counselling or usual care in improving couples’ sexual and psychosocial adjustment after diagnosis and treatment of localised prostate cancer	16 urologists in private clinics Public/private hospital in QLD 35 patients from a public service announcement in Australia	*N* = 189	Clinical: Newly diagnosed localised prostate cancer having radical prostatectomy or < 12 months post-surgery, heterosexual cohabitating couple r/ship Demographics: mean age men 62.70 (SD 6.80) mean age -women 59.78 (SD 7.38) Education: 65.1% of men completed some form of education/technical trade compared to 47.6% of women	Convenience	46.7% completed baseline assessments 76% completed all assessments 84% of couples completed 12 months follow-up	RCT	4	Questionnaires: Demographics: unstructured questionnaire. Sexual functioning). International Index of Erectile Dysfunction (IIEF). Female sexual Function Index (FSFI). Sexual supportive care needs Subscale of supportive care needs survey The psychological impact of erectile dysfunction- Sexual experience, Masculine self-esteem scale Relationship: The revised Dyadic Adjustment scale Program evaluation: open-ended questions	3-arm RCT-phone/ counselling support telephone-delivered in 6 (post-surgery recruitment) or 8 sessions (pre-surgery recruitment) Usual care: standard medical management, set of published education materials Nurse intervention: nurse counselling principles of cognitive behavioural & sexuality, behavioural homework, including expression of affection and non-demanding sexual touch, challenging negative beliefs about CaP ageing sexuality, helping the couple choose a medical treatment for ED and integrating into their sexual relationship Peer Intervention Shared personal experience, components include, psycho-educational experiences with surgery & recovery of ED management, managing and reviewing goals
Chambers et al. 2019 Australia	To investigate 5-year outcomes of couples-based intervention for men with localised prostate cancer	Extension of RCT Intervention (mail)	*N* = 240 Year 2 *N* = 236 Year 3 *N* = 235 Year 4 *N* = 215 Year 5 *N* = 201	Clinical: Completed RCT for newly diagnosed localised prostate cancer having radical prostatectomy or < 12 months post-surgery, heterosexual cohabitating couple r/ship Demographics: mean age men 62.70 (SD 6.80) mean age -women 59.78 (SD 7.38) Education: 65.1% of men completed some form of education/technical trade compared to 47.6% of women	Convenience	84% of men and 80.5% of partners completed 5-year assessment	Extension of RCT by invitation Mail validated self-report measures	4	Questionnaires: Demographics: unstructured questionnaire. Sexual functioning). International Index of Erectile Dysfunction (IIEF). Female sexual Function Index (FSFI). Sexual supportive care needs Subscale of supportive care needs survey The psychological impact of erectile dysfunction- sexual experience, masculine self-esteem scale Relationship: The revised Dyadic Adjustment Scale	3-arm RCT-phone/counselling support telephone-delivered in 6 (post-surgery recruitment) or 8 sessions (pre-surgery recruitment) Usual care: standard medical management, set of published education materials Nurse intervention: nurse counselling principles of cognitive behavioural & sexuality, behavioural homework, including expression of affection and non-demanding sexual touch, challenging negative beliefs about CaP ageing sexuality, helping the couple choose a medical treatment for ED and integrating into their sexual relationship Peer Intervention Shared personal experience, components include, psycho-educational experiences with surgery & recovery of ED management, managing and reviewing goals
Davison et al. 2005 Canada	To assess the feasibility of a prostate sexual rehabilitation clinic	Prostate Center Vancouver General Hospital	*N* = 90	Clinical: men with prostate cancer treatment-related sexual concerns. RP *N* = 121, EBRT *N* = 10, BT *N* = 10, WW *N* = 3, RP + salvage RT *N* = 5 Demographics: Mean age: 64.4 SD (7.82) Education: < high school-6.5%, high school -24.7%, 68% > High school level	Convenience	*N* = 155 approached *N* = 90 -58% responded partners *N* = 58 response 60% at 4 months	Quantitative study	2	Questionnaires: Demographics: unstructured questionnaire Sexual function: International Index of Erectile Dysfunction (IIEF-15). Feelings towards partner: The positive feeling Questionnaire (PFQ). Couples’ satisfaction: Satisfaction questionnaire (SQ) Couples satisfaction with treatment: The ED inventory of treatment satisfaction	A dedicated prostate sexual rehabilitation clinic Staffed by 2 trained Nurse Specialists in sexual health Clinicians conducted a detailed sexual assessment on the first visit Patients were provided information on available sexual aids i.e., oral medication, injections, vacuum devices, surgical implants Counselling about sexual repertoire to enhance the sexual experience Follow-up care
Grondhuis-Palacios et al. 2018 Netherlands	To explore who will provide sexual healthcare and when according to men with prostate cancer and their partner	Oncology Registration Leiden University	*N* = 253 *N* = 174 Partner	Clinical: Local disease *N* = 232, Regional node metastases *N* = 11, metastasised disease *N* = 8, TMN unknown *N* = 2. Treatment type: AS-N = 17, RP-N = 64, BT-25, IMRT-N = 60, IMRT + ADT-N = 71, ADT-N = 15, Other-N = 1 Demographics: Mean age: 69.3 SD 6,9 (range 45–89) Education: No qualification/elementary school 16–6.3%, Lower vocational 65–25.7% Intermediate vocational 56–22.1% Higher secondary 33–13.1% Higher education 81–32%	Convenience	Not reported	Cross-sectional survey	1	Questionnaires: Demographics: unstructured questionnaire Questionnaires designed by authors: 47 items sexual function before and after treatment, experience and satisfaction regarding current sexual healthcare and desired sexual management Partners questionnaire: 14 items about sexuality throughout their partner’s treatment & whether counselling in sexuality and /or relational matters would be appreciated	Questionnaire exploring the current situation of sexual healthcare and satisfaction of treatment options provided to men having treatment-related sexual dysfunction Investigate which healthcare provider is preferred and what is considered a suitable time for sexual counselling to commence
Karlsen et al 2017 Denmark	To assess the feasibility and acceptability of couple counselling and pelvic floor muscle training after surgery for prostate cancer	Department of Urology Rigshospitalet	*N* = 6 couples	Clinical: have a female sexual partner, undergone surgery within 3–4 weeks Demographics: Mean age 64.1 (58–72) Education: not reported	Convenience	*n* = 6 couples recruitment 14%	Single-arm trial	3	Questionnaires: Demographics: unstructured questionnaire Sexual function: International Index of Erectile Dysfunction (IIEF). Female sexual Function Index (FSFI)	Standard care-preoperative instruction in PFMT, regular outpatient visits with a physician referral to municipal rehabilitation. Medical treatment if not contraindicated- daily PDE5 inhibitor or on demand, Alprostadil pin or penile injections Pro Can intervention 6 × 1-h couples counselling (nurse certified in sexual counselling) On a need basis X1 group instruction in PFMT X3 Individual PFMT with physio (needs basis) & DVD PFMT for home training Couple Counselling Initiated as soon as a couple felt ready Semi-structured explorative and informing elements of sexual therapy Structured using the first 3 levels of the PLISSIT model
Karlsen et al. 2021 Denmark	To test the effect of the Pro Can intervention (couples counselling & PFMT) on sexual & urinary function after surgery for prostate cancer	Clinic of Urology Rigshospitalet, Copenhagen	*N* = 16 *N* = 19 Healthy controls	Clinical: men having radical prostatectomy nerve sparing and non-nerve sparing Demographics: Mean age 64.1 (58–72) Educational: not reported	Convenience	Not reported	RCT	3	Questionnaires: Demographics: unstructured questionnaire Patient-reported outcomes: Sexual function: International Index of Erectile Dysfunction (IIEF) Secondary outcomes: urinary function: Expanded Prostate Cancer Index Composite Short Form (EPIC -26) Sexual function: Female sexual Function Index (FSFI) Sexual distress: Female sexual distress scale Relationship function- Dyadic adjustment scale HRQOl: symptom checklist 92- anxiety Major depression Inventory General self-efficacy GSE	Two arms RTC 1:1 Usual treatment (both groups received this) Pre-surgery instruction in PFMT, regular tests for PSA, an outpatient visit to a physician, referral to municipal rehab (focusing on PFMT) treatment for ED was offered if not contraindicated with pde5 inhibitors, pin or penile injection of alprostadil Pro Can Intervention 6 1-h couples counselling with a certified sexual counsellor X3 individual instructions in PFMT complemented with a home video training program. Encouraged to complete at least 2 couples counselling and × 1 PFMT session Counselling indicated approx. 2–3 months after surgery and continued for 6 months discussing their feelings, relationship, intimacy and sex if interested introduced to sensuality training to increase their intimacy & desire without penetrative sex PFMT- credited Physio 3–4 months of study inclusion
Letts et al. 2010 Canada New Brunswick	To explore the impact of prostate cancer treatment on a broad range of aspects of men’s sexual wellbeing	Prostate cancer support Groups in 3 major centers in a small Canadian province	*N* = 19	Clinical: Radiotherapy *N* = 10, RP N = 9 1–5 years post treatment.50% reported physical health problems (diabetes, heart condition, heart surgery) Demographics: Mean age 65 (54–79) Age of diagnosis 49–74 Education: beyond high school diploma 45%	Purposive	Response rate *N* = 22 Refused to provide pre- and post-sexual function data *N* = 1 Cognitive difficulty *N* = 1 Unwell- *N* = 1	Qualitative study Interview 90 min (45–120) study	1	Questionnaires: Demographics: unstructured questionnaire Checklist potential post-treatment physical, emotional, and sexual symptoms rated 1–5 point scale used during the interview Interview Questions-Describe their pre & post treatment sexual desire, erections, sexual satisfaction, orgasm and frequency & type of sexual activities Nature and extent of changes on emotional impact & impact on partner Use & effectiveness of medical treatment	Qualitative Interviews to understand the men’s lived experience of the impact of Prostate cancer on aspects of their sexual well-being What changes to their sexual well-being What emotional changes do they experience Men’s perceptions of the impact of sexual changes on their partner’s sexual well-being What information is provided to men on the potential impact of sexual functioning and dealing with changes in their sexual functioning
Mehta et al. 2019 USA	To explore what patients and their partners want in interventions that support sexual recovery after prostate cancer treatment	Urology and radiation oncology out-patient departments at 2 academic medical center in the US Midwest & South represent Urban Suburban Rural	*N* = 14 *N* = 10 Partner	Clinical: *N* = 9 Radical Prostatectomy, *N* = 3 Radical Prostatectomy + RT N = 1 RT & ADT, N = 1 ADT only N = 3 same-sex partners Demographics: Mean age Patient-62 (51–84), Partner-63 (35–83) Education: Patient High school-N = 3–21% College degree-N = 10–71% Graduate degree N = 2–21% Education: partner High school-N = 2–20% College degree-N = 7–70% Graduate degree N = 2–20%	Convenience	Not reported	mixed methods study	1	Questionnaires: Demographics: unstructured questionnaire Diagnosis & treatment of CaP abstracted from patient notes Functional measures: Expanded Prostate Cancer Index Composite (EPIC) Sexual function: Female Sexual Function Index (FSFI). International Index of Erectile Dysfunction (IIEF) Interview Questions: 3 domains (i) Experience with prostate cancer treatment (ii) Support received/needed for sexual recovery (iii) Recommendations for an intervention that would aid sexual recovery after treatment	Sexual function assessments & validated instruments provided context for participants’ views expressed in the focus groups Qualitative component with focus groups & participants Interviews to understand their experiences with treatment side effects and support received & needed for sexual recovery
Miller et al. 2006 USA	To assess the prevalence and outcomes of erectile dysfunction therapy among long-term prostate cancer survivors and assess sexual motivation and patterns of ED therapy	Michigan Urology Center Controls University of Michigan Geriatric Center	*N* = 896 *N* = 112 Healthy Controls	Clinical: *N* = 665 Radical Prostatectomy (RP), Bilat NS 66%, Unilateral NS 12%, NNS 21% N = 147 3D-CRT, N = 84 BT, Time frame: 4–8 years post-treatment Demographics: Median age RP 67.2,3D-CRT 75.7, BT 70.4 Education- not reported	Secondary	72% overall response rate *N* = 650 (72.5%) Control response rate *N* = 74- (66%)	Quantitative survey Secondary analysis	1	Question to evaluate the current quality of unassisted erections: “How would you describe the current quality of your erections without the assistance of medications/devices during the past 4 weeks”? Sexual function: Expanded Prostate Cancer Index composite-26 Short form (EPIC-26) (EPIC). Item added “Overall, how big a problem has your sexual function or lack of sexual function during the past 4 weeks” Sexually motivated: if participants describe it as a big-moderate problem Indifferent: Small problem with current sexual function	Survey of the participants for the use of medications or devices and the frequency of use Interventions included: medications, sildenafil, intraurethral alprostadil, penile injection therapy, vacuum erectile device
Naccarato et al. 2016 Brazil	To evaluate the impact of group psychotherapy and the use of Pde-5i early in men who are undergoing surgery for CaP	Not reported	*N* = 53	Clinical: *N* = 53 preoperative patients undergoing NS and NNS Radical Prostatectomy NS- N = 26, NNS N = 21, Unilateral-N = 6 47% had prior ED before surgery Demographics: Mean age-61.84 (39–76) Education: Incomplete elementary school 37/53	Convenience	Response rate *N* = 56 Lost to follow-up *N* = 3	Prospective randomised control trial	2	Questionnaires: Demographics: unstructured questionnaire Individual interviews Questionnaire: developed by interviewers unvalidated (weekly) after surgery evaluating aspects of intimacy with partner & satisfaction with sex life Quality of Life: Short form Health Survey Questionnaire (SF36) Sexual function: International Index of Erectile Dysfunction (IIEF)	Group psychotherapy & Pde5 inhibitors Group 1: Control Group 2: Group psychotherapy Group 3: Lodenafil 80 mg per week Group 4: Group psychotherapy Lodenafil 80 mg weekly
Nelson et al. 2019 USA	To assess the feasibility of psychological intervention based on acceptance and commitment to utilise penile injections for penile rehabilitation	Sexual Medicine Program Memorial Sloan Kettering Cancer Center	*N* = 53	Clinical: RP (open, robotic) within 9 months, good ED function pre-op, Demographics: mean age ACT-ED- 60 (SD 7.5) EM- 61 (SD 7.3) Education: College degree or higher total 73%	Convenience	89% *N* = 47 completed interventions ACT- N = 22 EM-N = 25 81% 83% *N* = 44 completed all 4 monthly study ACT-N = 21 EM- N = 23 8 months 60% ACT-N = 15 EMN = 17	Pilot randomised RCT	3	Questionnaires: Demographics: unstructured questionnaire Primary outcome: injection use, syringes used to count at 4- & 8-month visit Secondary study outcomes several self-report measures Satisfaction of using penile injections Erectile dysfunction Inventory of treatment satisfaction (EDITS) Sexual self-esteem & confidence: Sexual self-esteem and relationship questionnaire (SEAR) Sexual bother (SB) assessed the subscale of Prostate health-related quality of life. Depression is assessed using the Center for Epidemiological studies depression revised scale. Prostate cancer treatment regret: 5-item questionnaire scale to reflect on the decision of selecting surgery as their treatment for CaP Sexual Function: EFD subscale of International Index of Erectile Dysfunction (IIEF)	Psychological intervention Randomised 1:1 Standard care (SC) initial visit to sexual medicine clinic (6–24 weeks post-surgery), Penile rehabilitation concept & Injection training 2 sessions (1 h), phone calls to titrate the dose, 4 months f/up Enhanced Monitoring: (EM) 7 phone contacts at the same time interval as ACT-ED Acceptance & commitment therapy (ACT) for ED Acceptance & willingness to experimental exposure commitment, Clinical psychologist 3 brief (5–10 min) phone calls progressed until 4 weeks apart Last ACT-ED in person
Obrien et al 2010 UK	To determine the Unmet psychosexual needs of prostate cancer patients during follow-up treatment	3 General practice settings North Wales East Lothian Thames Valley	*N* = 35 *N* = 18 Partner	Clinical: treatment curative, hormonal, monitoring and by whom primary, secondary or shared care. Follow-up range 9 months -14 years post-treatment Demographics: age 59–82 Education: not reported	Purposive	45 patients approached reasons not recorded for participants that declined study	Qualitative study	1	Exploratory interviews: Open non-directive questions Discussion with consultations: Identifying men’s unmet psychosexual needs, Lack of rapport with staff, Living with side effects: Concealment, resistance & acceptance of psychosexual problems, unmet psychosexual needs of older patients, partners’ psychological needs	Postal Invitations to patients and/or partners could be included Exploratory interviews were conducted using a topic guide Including; diagnosis, current treatment, and follow-up care
Pillay et al. 2017 Australia	To Explore QOL, psychological functioning & treatment satisfaction of men who have had penile prosthesis after radical prostatectomy	2 Private urology practices	*N* = 71 *N* = 43 Partner	Clinical: Penile implant following RP, the average time since surgery 933 (SD 466) Demographics: Mean age 63.2 men, partners 59 Education: At most school education All men-25% Men & partner-29% Partners -40% TAFE/TRADE All men-19% Men& partner-24%% Partners -2% Undergraduate Degree All men-29% Men& partner-24% Partners -28% Post Graduate training All men-26% Men& partner-22% Partners -30%	Convenience	72.4%	Cross-sectional Retrospective study	1	Questionnaires: Demographics: unstructured questionnaire Patient questionnaires: Prostate-specific Quality of life scales: Clark scales The expanded Prostate Cancer Index Composite Short Form (EPIC-26) Depression & anxiety: Generalized Anxiety disorder-7 (GAD-7), Patient Health Questionnaire-9 (PHQ-9) Sexual Dysfunction: The erectile dysfunction Inventory of treatment satisfactory (EDITS) Sexual self-esteem & confidence: Sexual self-esteem and relationship questionnaire (SEAR) Partner Questionnaire Generalized Anxiety disorder-7 (GAD-7), Patient Health Questionnaire-9 (PHQ-9) PHQ-9 The erectile dysfunction Inventory of treatment satisfactory (EDITS)-partner version Sexual self-esteem & confidence: Sexual self-esteem and relationship questionnaire (SEAR) General feedback questions about penile prosthesis process-24 questions opened ended developed by the research team Partners answered 11 questions	Penile Prosthesis post prostatectomy surgery
Shrover et al. 2012 USA	To evaluate the effect of internet-based or traditional sexual counselling for couples after localised prostate cancer treatment	MD Anderson Cancer Center Texas	*N* = 115 couples	Clinical: treatment for localised prostate cancer Either RP: FF-N = 70, WEB1-N = 68, WEB2-N = 84. RT: FF-N = 30, WEB1-N = 32, WEB2-N = 16 Demographics: men age FF-64 (SD + -8), WEB1-64 (SD + -7), WEB2-64 (SD + -8), Education: high school FF-N = 8, WEB1-N = 5 WEB2-N = 9. Some college FF-N = 20, WEB1-N = 15, WEB2-N = 30. College degree FF-N = 43	Convenience	Final 51 couples completed 4 sessions-dropout with rates 39%	RCT	5	Questionnaires: Demographics: unstructured questionnaire Sexual function: International Index of Erectile Dysfunction (IIEF). Female sexual Function Index (FSFI) Current distress: Brief symptom inventory-18 (BSI-18) Relationship satisfaction: Dyadic Adjustment Scale (A-DAS)	Internet-based/traditional based Sexual counselling Randomised (adaptively) 3 months wait for the list (WL) After the waiting period, WL participants were randomised to FF or WEB1 Face-to-face format (FF) 3 sessions over 12 weeks (90 min, 50–60- 2,3) Internet-based format (WEB1) immediate intervention group & email contact with the therapist Second Internet-based group (WEB2) for participants who lived too far away examined r/ship website use and outcomes
Wittmann et al. 2013 USA	To assess the feasibility of a one-day couple group intervention to Increase couple’s awareness or resources for sexual recovery for treated men with prostate cancer and their partners: A pilot study	Mid-western University comprehensive cancer center	*N* = 52 *N* = 26 partner	Clinical: Patients treated with surgery for CaP and their partners. Men three years post-surgery Demographics: Mean age: Patients-67 (SD = 6.4) Partners -65 (SD 6.8) Education –medium length-Men *N* = 16 years Partners *N* = 14.5 years	Purposive	Response rate 88.5% 3 months-75% 6 months 63.5%	One sample design	Time points Pre-/post time point 3 months & 6 months post	Questionnaires: Demographics: unstructured questionnaire Sexual function: The erectile dysfunction help-seeking questionnaire The protective buffering scales Satisfaction: Satisfaction questionnaire non-validated The sexual information and recovery activities (non-validated) General Analytic Strategy	One day Couple retreat Biopsychosocial psycho education group intervention Retreat morning session Developed by a Multidisciplinary team Education component provided by healthcare professionals Afternoon session Participants discussed their experience of sexual recovery in separate patient & partner groups of 8 each facilitated by a master’s level social worker certified as a sex therapist or urologic nurse who Discussion of themes from groups
Wittmann et al. 2015 USA	To assess the feasibility of the development of a conceptual model for couples’ sexual recovery after prostate cancer surgery	Mid-western academic cancer center	N = 20	Clinical: Men having RP as primary treatment N = 20 heterosexual couples, N = 1 same sex Stage T1c-N = 17, T2a-N = 2, T2b-N = 1 NS-N = 18, Partial N = 2 Preoperative ED mean 74.4 (SD 25.1). Postoperative ED mean 46.5 Demographics: men: mean age 60.2 Partners’ mean age is 57.6 Education: educated beyond high school Patients N = 70%, Partners N = 50%	Convenience	Response rate *N* = 20 eligible 8 couples refused for various reasons secondary analysis	Qualitative	2	Questionnaires: Demographics: unstructured questionnaire Sexual and urinary domains: Men Expanded Prostate Cancer Index Composite (EPIC) Short form. Female Sexual Function Index (FSFI) Qualitative assessments: based on literature review & researchers’ clinical experience Preoperative: Are you aware you will experience side effects that affect urinary control and the ability to have erections? What are your thoughts about those? Postoperative: Can you tell me about your experience of recovering your sexual relationship since surgery?	Couples’ experiences were assessed by semi-structured one-hour couple interviews followed by brief individual interviews Interview guides were based on reviewed literature and the researcher’s clinical experience
Wittmann et al. 2015 USA	To assess potential preoperative barriers to couples’ sexual recovery after radical prostatectomy for prostate cancer	Mid-western academic cancer center	*N* = 28 couples	Clinical: Men who have chosen RP. Stage T1c-N = 1b N = 2, T1c-N = 20, T2a-N = 4, T2b N = 2 Mild erectile function pre-op Demographics: Patients Mean age 62.2 (50–74), Partners 58.4 (38–70) Education: Patient Beyond high school- 20- 71%, Partners – 17- 61%	Convenience	Eligible couples *N* = 108 Sample *N* = 28 26% response rate	Prospective Mixed method design	1	Questionnaires: Demographics: unstructured questionnaire Couple adjustment: Dyadic Adjustment Scale (A-DAS) Couple communication: Protective buffering scale Sexual function: The Expanded Prostate Cancer Index Composite Short Form (EPIC-26), Sexual satisfaction: Male; The Sexual Experience Questionnaire, Short form. Female Sexual Function Index (FSFI) Qualitative assessments: based on literature review & researchers’ clinical experience Couples’ questions: As you get ready for surgery, can you describe your thoughts about it and any concerns? How do you think you and your partner will cope emotionally and with sexual changes?	A couple-focused interview followed by brief interviews with patients and partners separately to identify potential barriers to sexual recovery before radical prostatectomy
Wootten et al. 2014 Australia	To assess the feasibility and usability of an online psychological intervention for men with prostate cancer	Urology Practices Melbourne	*N* = 64	Clinical: Treatment RP -N = 62.46%, BT-N = 2%, in last 5 years, Time since diagnosis 27 months Demographics: Mean age 64 Education -University degree 41%, Trade 17% Post school education12%	Convenience	Eligible *n* = 75 11 excluded did not fit inclusion criteria *n* = 64 attrition 31%	Feasibility questionnaires	Baseline Completion of intervention	Questionnaires: Demographics: unstructured questionnaire Psychological distress: Depression and anxiety Stress scales (DASS-21) Sexual function: International Index of Erectile Dysfunction (IIEF) The questionnaire developed: to assess participants’ satisfaction Open-ended questions to express their opinion of the best or worst part of the intervention	Online intervention 6 self-directed modules based on CBT principles & worked through sequentially Designed for single men & men in an intimate relationship (road map of participant’s journey Moderated online which participants could post comments questions or share their experiences forum
Wootten et al. 2016 Australia	To investigate whether an online psychological intervention can improve the sexual satisfaction of men following treatment for localised prostate cancer	Self-referral following invitation to join the study by Urologist, advertisements in newsletter, website and via post cards	*N* = 142	Clinical: Localised CaP having/have curative treatment within 5 years, Time since diagnosis 3.5 years Demographics: Mean age 61 SD 7 (42–82) Education -not reported	Convenience	Completion rate 87% Week 5 73% post-treatment 3 months 66% 6 `months 51%	RCT	4	Questionnaires: Demographics: unstructured questionnaire Psychological distress: Depression and Anxiety Stress Scales (DASS-21) Sexual function: International Index of Erectile Dysfunction (IIEF) Quality of life: The prostate cancer-related Quality of Life Scale (PCR-QOL) Self-report: Use of sexual aids	Online psychological intervention for prostate cancer 3 interventions Group1 -MRA program- self-guided Group 2- MRA + forum Two moderated forums that were Group 3 access to moderated forum only Allocation 1:1:1
Yiou et al. 2013 France	To investigate the sexual quality of life in female partners of men using IAI after RP	Uro Oncology Department	Sample size: *N* = 104 Couples	Clinical: Laparoscopic RP- NS & NNS Bilateral NS- N = 78, Unilateral NS-N = 14 NNS- N = 12 Demographics: Mean age Men: 62.3 m (SD6.1) Female 59.8 (SD 7.3) Education: not reported	Convenience	*N* = 152 couples eligible abandon IAI *N* = 29 treatment due to pain, lack of efficacy *N* = 19	Cross-sectional retrospective longitudinal study	1	Questionnaires: Demographics: unstructured questionnaire Individual interviews Sexual function: Male, International Index of Erectile Dysfunction (IIEF-15). Erection Hardness Score (EHS) Continence: International Consultation on Incontinence questionnaire (ICIQ) Urinary function questionnaire (UCLA -PCI) Pain score: Visual analog scale (VAS) Impact of ED: Female Index of sexual life (ISL) Global life satisfaction (GLS) GLS- 2 items Questionnaire Has your sex life been disrupted by excessive tiredness, psychological distress, disease, gynaecological problems or lack of availability?	A post-RP-Sexual rehabilitation program, 1 month after RP Weekly f/up then 6 monthly participants use of IAI -Intracavernous alprostadil was monitored after 1 year of use Female partners completed questionnaires to assess their sexual quality of life

**Key**: *CaP* cancer of the prostate**,** *RP* radical prostatectomy, *RT* radiotherapy, *NS* nerve sparing, *NNS* non-nerve sparing, *EPIC* Expanded Prostate Cancer Index Composite, *IAI* intracavernous alprostadil injection, *IIEF* International Index of Erectile Function, *EHS* Erection Hardness Score, *FSFI* Female Sexual Function Index, *SF36*, Short Form Health Survey Questionnaire, *BSI-18* Brief Symptom Inventory, *A-DAS* Dyadic Adjustment Scale, *DASS-21* Depression & Anxiety Stress Scales, *PFQ* Positive Feelings Questionnaire**,** *SQ* Satisfactory Questionnaire, *EDITS* Erectile Dysfunction Inventory of Treatment Satisfaction, *EPIC-26* Expanded Prostate Cancer Index Composite Short Form Index, *GAD-7* Generalised Anxiety Disorder, *PHQ-9* Patient Health Questionnaire, *ICIQ* International Consultation on Incontinence Questionnaire, *UCLA-PCI* UCLA Prostate Cancer Index, *VAS* visual analogue scale, *ISL* Index of Sexual Life, *GLS* General Life Satisfaction, *SD* sexual drive, *DASS21* Depression & Anxiety Scale Short Version, *PFMT* pelvic floor muscle treatment, *PEID-SE* Psychological Impact of Erectile Dysfunction – Sexual Experience, *SEAR* Self-Esteem and Relationship Questionnaire in Erectile Dysfunction, *EDITS* Evaluating Satisfaction with Treatments for Erectile Dysfunction, *SCL-92* Symptom Check List 92, *MDI* Major Depression Inventory, *GSE* General Efficacy Scale, *GMSEX* Global Measure of Sexual Satisfaction, *HADS* Hospital Anxiety and Depression Scale, *FFMQ-SF* Five Facet Mindfulness Questionnaire Short Form, *PLISSIT* Permission, Limited Information, Specific Suggestions, Intensive Therapy (model of sex therapy)

The studies included randomised control trials (*n* = 6), other quantitative studies (*n* = 5), qualitative studies (*n* = 2), and mixed methods studies (*n* = 5). Twelve of these studies involved patients and partners, while five included patients only [20, 22, 35–37]. Overall, the methodological quality of the studies was creditable, except for three studies [36, 38, 39] which did not meet all the quality assessment criteria (see Table 2).

**Table 2:**
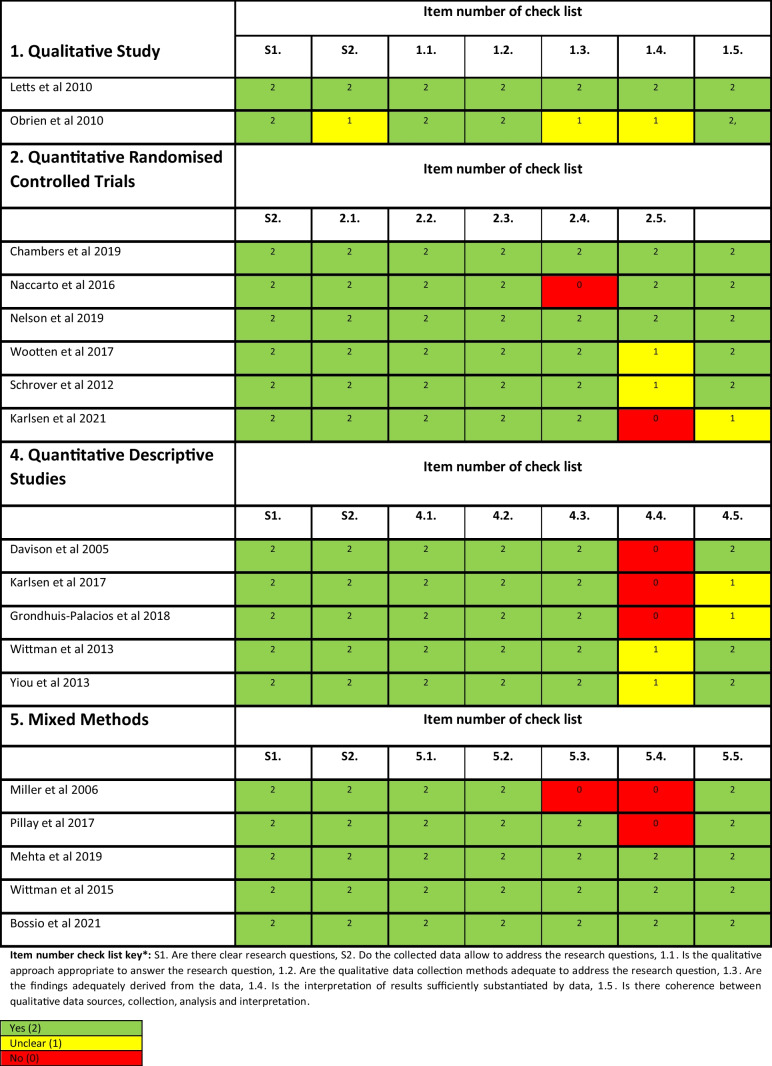
Quality appraisal of primary studies

There were two main classifications within existing interventional research for sexual well-being, which were either medical or pharmacological (*n* = 4) and psychological (*n* = 7). The remaining studies represented the patients’ and intimate partners’ perspectives on sexual well-being interventions (*n* = 7) [20, 21, 38–42]. All studies represented men diagnosed with prostate cancer treated with surgery, radiotherapy, androgen deprivation therapy, active surveillance, and their partners. Only three studies included representation from same-sex couples. A total of 13 same-sex couples provided insights into their unique needs and preferences for sexual well-being interventions [35, 40, 41]. Two studies [40, 41] investigated what couples wanted in terms of interventions that support sexual recovery [35, 40, 41]. Wootten (2017) developed an online psychological intervention for men with prostate cancer [35, 40, 41].

## Qualitative experiences

Overall, three themes emerged which related to (1) communication (with the couple, healthcare professionals, and peer support), (2) educational and informational needs, and (3) timing and delivery of the interventions. In addition, within each of these themes, barriers and facilitators were identified.

### Theme 1: Communication

This overarching theme included three sub-themes related to communication between (a) patient and intimate partner, (b) healthcare professionals, and (c) peers. Several studies identified a lack of communication between the couple as an initial barrier to accessing assistance with sexual well-being interventions [21, 34, 40–42]. Communication was problematic for couples due to a lack of language knowledge or discomfort with discussing sexual issues. Often, couples relied mainly on non-verbal prompts [41]. The discomfort with discussing sexual issues was a consistent finding in three studies [19, 20, 39]. Couples had trouble in initiating a conversation to discuss intimacy, which compounded further complexities in accessing treatment options for sexual well-being recovery [39]. The issue of communication often led couples to evade the topic and deflect their thinking to other areas of recovery [19].

Communication with healthcare professionals (HCP) was identified as a challenge for patients and partners, particularly discussing sexual health needs [21, 34, 40, 43, 44]. One study [19] reported healthcare professionals also experienced trepidation in initiating the topic of sexual well-being with patients [19]. Patients expressed that the onus was on them to initiate the conversation with healthcare professionals and often felt embarrassed to discuss their sexual concerns [21]. Many patients and partners consequently were left with feelings of stress, frustration, and disappointment [21]. In addition, some patients found healthcare professionals dismissive, assuming older men did not require such information, and patients commented that there was no continuity of care by seeing several doctors in clinic and they had to repeat their sexual issues [20, 21, 40]. Patients described that some healthcare professionals would focus on cancer control rather than directing consultations to the long-term impacts of sexual dysfunction on quality of life [40]. The impacts of sexual dysfunction in gay men’s sexual experiences were often unmet because their experiences were different from heterosexual couples, so healthcare professionals were reticent to engage in a conversation with them [40].

The impact of peer support was valued among patients and their partners [15, 34, 35, 40, 45, 46]. Likewise, peer support was recognised as beneficial in providing the opportunity for patients and partners to discuss and normalise their treatment and sexual well-being recovery [35]. In addition, two studies [15, 40] reported that peer support which provided practical coping advice and assisted with navigating both physical and psychological needs was helpful [15, 40].

Two studies [45, 46] reported the benefits of group peer support interventions. Wittman’s (2013) study involved a one-day retreat with peers and identified that the peer support intervention improved satisfaction between couples for at least six months following the intervention by facilitating open dialogue [46]. The Bossio (2021) study explored a mindfulness group–based intervention. Group interventions allowed patients and their partners to experience acceptance-based communication around intimacy and agreement of a “new sexual normal” [45]. These interventions improved communication between couples and promoted sexual intimacy beyond penetrative intercourse [40].

Several studies [20, 39, 40, 44, 46] identified facilitators which promoted both patient and partner sexual well-being discussions and facilitated communication with healthcare professionals. Enabling open communication with healthcare professionals provided the space to develop a mutual understanding of the expectation of treatments, with realistic expectations of the success of various sexual well-being interventions to minimise the distress of failure [44]. Couples described improved relationship satisfaction when they were given the opportunity to explore different strategies and discuss sexual changes over time [39]. Promoting open, safe, and non-judgemental dialogue between couples and healthcare professionals enabled the timely opportunity to discuss sexual changes. This opportunity provided a positive experience for patients and partners in that their concerns were validated [20, 40].

### Theme 2: Educational and informational needs

Several studies [15, 20, 22, 39–41] identified barriers to accessing information and education for couples concerning unmet sexual health needs. Patients had difficulty in timely access to healthcare professionals to provide education and information [22, 39]. A lack of informational support was a problem for couples, particularly in the pre- and postoperative phases [20, 40]. Many participants reported a lack of information about the side effects of prostate cancer treatment, specifically regarding the sexual and emotional changes which were likely to occur [20, 40]. Inadequate information led many couples to express frustration, disappointment, and distress which presented a barrier to accessing or using sexual interventions [20, 40, 41]. In contrast, one study [15] reported that patients and partners felt they received copious amounts of information leaving them with feelings of information overload, which was perceived as a barrier [15].

Five studies [15, 39, 45, 47] explored the benefits of providing education for patients and their partners. Findings identified that education should address specific supportive care needs such as self-management of side effects of treatments [15], addressing realistic patients’ expectations [39, 47], and targeting support through education for the couples’ sexual functioning [45]. Two studies [43, 44], using medical interventions (penile prosthesis and intracavernosal injections), identified partner inclusion to be essential in the delivery of sexual healthcare. Including the partner provided support for the couple with a mutual understanding of the intervention and the opportunity to express their concerns [43, 44]. Three studies [35, 41, 46] identified that educational support should focus on couples’ emotions and adaption, which should include grief and loss of their sexual function.

### Theme 3: Timing and delivery of interventions

Three studies [21, 38, 40] recommended a structured approach to assessing sexual well-being needs to optimise the timing and access to interventions. Healthcare professionals should explore sexual well-being needs at regular intervals from the point of initial presentation and continue throughout their treatment across the entire cancer care continuum [45]. The timing and delivery of sexual well-being interventions were essential to patients and partners as they required time to assess their needs. Some patients wanted to engage early in their treatment process, whereas others preferred to wait. One patient described that he would like access to a website that has the entire recovery process mapped out, using video, people to talk to, and an outlet for emotional support [40]. Two studies [40, 41] suggested that three months post-treatment was the optimal timing for initiating sexual well-being interventions in the context of their individualised couple-based intervention. These studies suggest that this time point may allow couples to adjust to side effects from treatment and time for the patient to grieve their loss of sexual function [40, 41]. However, O’Brien et al. recommend an individualised approach, with regular psychosexual assessment by healthcare professionals at routine appointments to facilitate timely and accessible sexual well-being recovery interventions [21], underscoring that one size does not fit all [35, 40, 46].

## Patients’ experience of participating in sexual well-being interventions

Several studies [35, 41, 45–47] reported on interventions to improve or enhance sexual recovery. Sexual side effects from treatment include erectile dysfunction, climacturia (involuntary loss of urine at orgasm), anorgasmia (unable to obtain orgasm), urinary or faecal incontinence, penile shortening, and loss of sexual desire [45]. Sexual recovery involved engaging couples in interventions to improve sexual intimacy. Engaging couples included education and support to encourage effective communication, promote awareness of sexual well-being resources, and provide strategies for coping with the physical and emotional challenges of treatment side effects [35, 41, 45–47]. Patients and their intimate partners preferred interventions with a component of peer support and delivered to their individual needs within a suitable time frame [15, 40, 45].

Two broad categories of sexual well-being interventions were identified across the 18 studies. The interventions included medical or pharmacological interventions with the addition of a psychological component [15, 22, 34, 36, 37, 40, 43–46]. There was a diversity among the interventions regarding composition, timing, and outcomes, and most of the study outcomes focused on erectile function and intervention compliance [15, 34, 37, 44]. The studies included couples’ sexual recovery and satisfaction from the interventions [15, 34, 40, 45, 46].

### Erectile function

Several studies reported erectile function using the International Index of Erectile Function (IIEF) [22, 36, 47] or the Expanded Prostate Cancer Index Composite (EPIC). The IIEF measure is a 15-item self-report instrument of male sexual function, including sexual desire, satisfaction, erectile function, and orgasm. The score range is between 1 and 25. Severe erectile dysfunction is rated 1–7, moderate 8–12, and mild 17–21 and functional erections are between 22 and 25 [48]. The Naccarato (2016) study indicated that 47% of men had erectile dysfunction in the mild range (14–19) at baseline [22]. A similar finding in Davison’s (2005) cohort (*n* = 155) indicated an overall score on IIEF (19.98) at baseline which indicated erectile dysfunction [38]. Three studies [40–42] utilised the Expanded Prostate Cancer Index Composite (EPIC). The Expanded Prostate Cancer Index Composite is a prostate cancer health-related quality of life instrument that measures general symptoms relating to urinary, bowel, sexual, and hormonal issues to provide a comprehensive subjective assessment of patients having treatment for prostate cancer [49]. The scale for erectile dysfunction (ED) using the Expanded Prostate Cancer Index Composite is severe ED (0–33), moderate ED (34–45), mild ED (61–75), and no ED (< 75). Mehta’s (2017) study indicated that men (*n* = 14) had a mean EPIC score of 20.8 (8.3–53.6) at baseline indicating severe erectile dysfunction [40]. Two studies reported on men preoperatively [41, 42]. The Wittman & Carolan (2015) study (*n* = 28) reported the average sexual function score on the EDITS was 76.6, indicating that they had mild to no ED. However, a majority of these men used phosphodiesterase to assist the quality of their erections. Similarly, Wittman & Northouse (2015) identified preoperative erectile dysfunction in men (*n* = 20) experiencing mild ED mean score of 74.4 (SD 25.1). However, this deteriorated to 46.5 (SD 25.1) three months post-surgery which was statistically significant (< 0.0001) [42]. The inability to achieve an erection suitable for penetration following radical prostatectomy is a well-documented symptom. Damage of the cavernous nerves is thought to be a major cause and recovery may take from 18 to 24 months to recover [50].

### Partners’ experience

Two studies [43, 44] focused on erectile function and included the partner’s experience. Pillay’s study [43] examined the quality of life, psychological functioning, and treatment satisfaction of men undergoing penile prosthesis insertion following radical robotic prostatectomy. Overall, patients and partners had positive experiences with treatment satisfaction and sexual relationship following penile prosthesis during this intervention [43]. In contrast, Yiou’s (2013) study aimed to investigate the sexual quality of life in women whose partners were using intracavernosal injection therapy. This retrospective longitudinal study investigated men and their female partners one year following radical prostatectomy while men were using penile injections. The women’s sexual life satisfaction significantly correlated with the partners’ response to erectile function (*r* = 0.41, *p* < 0.00002) and intercourse satisfaction (*r* = 0.27, *p* < 0.005) [44].

### Psychological interventions

Four randomised control trials tested sexual well-being interventions on erectile function as the primary outcome and included a psychological component to the interventions [15, 22, 34, 37]. The psychological component was conducted by a clinical psychologist or counselling, which involved a nurse or sexual counsellor or peer support [15, 34]. The psychological intervention consisted of coaching and support for men and their partners either delivered individually or in a group format. At baseline, there were no significant differences in utilisation of treatments for erectile dysfunction (*G*^2^ = 1.06); at 12 months post-intervention, there was a meaningful increase in overall use of medical treatments among the groups (*G*^2^ = 9.77, *p* = 0.008). The peer intervention group was 3.14 times and nurse intervention group was 3.67 times more probable to use medical treatments for erectile dysfunction than those in usual care group [15]. Mainly when psychological intervention or counselling support was offered, it promoted better acceptance of sexual well-being interventions [15, 22, 34, 37].

### Satisfaction of interventions

Four studies [35, 41, 45, 47] identified that peer and group support interventions had greater sexual satisfaction rates for couples. Sexual satisfaction related to patient’s and partner’s confidence in navigating sexual dysfunction pathways, ease of the sexual conversation, and a focus on the return of intimacy and not just erectile function [35, 41, 45, 47].

## Discussion

The sexual well-being interventions identified in this review varied in content and methodology. The interventions were unimodal such as penile injections, phosphodiesterase medication, or penile implants, or multimodal, including the addition of psychological support such as counselling, group therapy, and mindfulness.

Sexual well-being concerns are a prominent unmet need identified throughout the literature [13, 51]. Various barriers to accessing sexual well-being interventions have been noted by patients and their partners and include communication, timely support from healthcare professionals, and consistent support through their cancer continuum [13, 51]. This integrative review examined patients’ and partners’ experiences of accessing sexual well-being interventions. This integrative review has shed light on the paucity of studies in other genitourinary cancers. Sexual well-being has been a significant unmet need in other GU studies in testicular cancer [8], bladder cancer [10], kidney cancer [9], and penile cancer [11]. Although this review aimed to understand the experiences of men and their partners with GU cancers, the literature comprised only prostate cancer studies, which is an important observation.

Despite this review containing entirely prostate cancer studies, sexual health remains one of the most common unmet needs among these patients into survivorship. A publication by Maziego (2020) reported on the long-term unmet supportive care needs of prostate cancer survivors 15 year following diagnosis. The salient findings identified that men find communicating about sexual needs a challenge and particularly gaining healthcare professionals’ help and support was a moderate/severe need [51]. Similar findings have been reiterated within this systematic review identifying communication with healthcare professionals and initiating a conversation about sexual health needs is a barrier for patients.

In developing future sexual well-being interventions, the healthcare professionals must ensure that the patients’ unmet sexual needs are identified and addressed [8, 10]. One recommendation is to address the patients’ unmet needs by completing a biopsychosocial screening at the time of their clinic review. The biopsychosocial screening assesses the patient’s physical, functional, and psychological needs and prioritises the individual’s needs [52]. Identification of patients’ unmet needs early in their cancer care has the potential to provide a more positive outcome in addressing and meeting their needs [51].

Communication with healthcare professionals (HCP) was identified as a challenge for patients and partners, particularly discussing sexual health needs [21, 34, 40, 43, 44].

Healthcare professionals should have a responsibility to engage with patients in sexual health discussions. However, the evidence reveals that they experience barriers such as lack of knowledge and lack of training in this field [54]. A recent review has identified the need for training in sexual health communication for healthcare professionals [54]. The optimal mode of delivery for this education should have a role in both undergraduate and postgraduate programmes, and one option might be in role-play approaches to learning integration [54]. Tertiary education institutions may have a role to improve sexual health communication for healthcare professionals by including training in their core curricula. This will ensure that preparation for the healthcare professional is adequately addressed to assess patients’ sexual needs.

Healthcare professionals and particularly nurses are in the optimal role to ensure that patients can discuss their sexual needs. In addition, it is crucial to maintain open lines of communication and active listening between the healthcare professional, the patient, and the partner. Open communication is fundamental when discussing the potential impact of treatments on their sexual well-being recovery and providing tailored education to patients and partners [5].

An interdisciplinary approach involving partners, peers, and other healthcare professionals by providing information/education evidence-based care will assist in addressing this unmet need. Continual assessment and management of patient’s sexual health concerns at clinical visits will provide timely treatment and evaluation of physical and psychological needs [46, 53].

This review has identified that patients benefited from sexual well-being interventions but many articulated difficulties with initiating the topic with healthcare professionals and timely access to the interventions. In developing strategies to promote timely access to evidence-based information and support, it is crucial to continue to gain an understanding of patient’s experiences and sexual health needs across the cancer continuum and how best healthcare professionals can support them.

### Limitations of the study

Although a structured and rigorous process was instigated throughout this integrative review, some limitations were noted. There is first a language bias noted from limiting studies to the English language, and this could mean that some critical studies may not have been included. However, the studies included represented various countries. Several key challenges were identified in this review; the different methodologies used in the studies made the synthesis of evidence challenging. Some studies contained small participant numbers; notably, all the studies involved patients with a diagnosis and treatment for prostate cancer. There was also a deficit in studies involving female GU patients’ experiences. These findings may not extrapolate into other GU cancer groups as the treatment, side effects, and recovery time differ among GU cancers. However, this review has presented an overview of men and their partners’ barriers and facilitators in accessing and using sexual well-being interventions and their experiences in sexual well-being recovery.

## Conclusion

This review contributes evidence of sexual concerns of men and their partners from diagnosis, treatment, and into survivorship. It has provided valuable insights into prostate patients’ and partners’ preferences and experiences when accessing or using sexual well-being interventions. Lack of continuity of care and timing of the interventions were identified as important findings. There was an overwhelming paucity in the literature for other GU cancers with sexual well-being interventions and limited representation of the LGBTQ + population. Further research is urgently required in the non-prostate GU cancer population.

## Supplementary Information

Below is the link to the electronic supplementary material.

Supplementary file1 (DOCX 14 KB)

Supplementary file2 (DOCX 24 KB)

## Data Availability

All generated or analyzed data during this study have been included in this published article.
