# Supplementary material for: Experiences of sexual well-being interventions in males affected by genitourinary cancers and their partners: an integrative systematic review
Source: Support Care Cancer. 2023 Apr 14;31(5):265. doi: 10.1007/s00520-023-07712-8 (PMC10104925; doi:10.1007/s00520-023-07712-8)
Supplement: Supplementary file 1 — Supplementary file1 (DOCX 14 KB) [file 520_2023_7712_MOESM1_ESM.docx]

**Supplementary Table 1- Database searches**

**Search strategy**

Five databases and one register were searched on 19 July 2021 to identify relevant studies (APA PsycINFO (EBSCOhost), CINAHL (EBSCOhost), Cochrane Database of Systematic Reviews, Cochrane Central Register of Controlled Trials, Medline (EBSCOhost), and Scopus. Limiters were applied to each database search for publication year (1997-) and for studies published in English. Searches returned a total of 2,509 results. Search terms and number of results by database:

**APA PsycINFO (183)**

((“sexual well-being” OR “sexual wellbeing” OR “sexual health” OR “sexual satisfaction” OR “sexual function*” OR “sexual wellness”) AND (cancer N5 (genitourinary OR prostate OR renal OR bladder OR testicular OR penile)) AND (intervention* OR treatment* OR therap* OR program* OR strateg*) AND (“lived experience*” OR “patient reported outcome*” OR “self-reported outcome*” OR “quality of life” OR “mental wellbeing” OR satisfaction OR dissatisfaction OR “psychological impact*” OR “body image” OR “self-image” OR ((relationship OR partner) N5 satisfaction)))

**CINAHL (276)**

((“sexual well-being” OR “sexual wellbeing” OR “sexual health” OR “sexual satisfaction” OR “sexual function*” OR “sexual wellness”) AND (cancer N5 (genitourinary OR prostate OR renal OR bladder OR testicular OR penile)) AND (intervention* OR treatment* OR therap* OR program* OR strateg*) AND (“lived experience*” OR “patient reported outcome*” OR “self-reported outcome*” OR “quality of life” OR “mental wellbeing” OR satisfaction OR dissatisfaction OR “psychological impact*” OR “body image” OR “self-image” OR ((relationship OR partner) N5 satisfaction)))

**Cochrane Database of Systematic Reviews (29)**

((“sexual well-being” OR “sexual wellbeing” OR “sexual health” OR “sexual satisfaction” OR “sexual function*” OR “sexual wellness”) AND (cancer NEAR (genitourinary OR prostate OR renal OR bladder OR testicular OR penile)) AND (intervention* OR treatment* OR therap* OR program* OR strateg*) AND (“lived experience*” OR “patient reported outcome*” OR “self reported outcome*” OR “quality of life” OR “mental wellbeing” OR satisfaction OR dissatisfaction OR “psychological impact*” OR “body image” OR “self image” OR ((relationship OR partner) NEAR satisfaction)))

**Cochrane Central Register of Controlled Trials (249)**

((“sexual well-being” OR “sexual wellbeing” OR “sexual health” OR “sexual satisfaction” OR “sexual function*” OR “sexual wellness”) AND (cancer NEAR (genitourinary OR prostate OR renal OR bladder OR testicular OR penile)) AND (intervention* OR treatment* OR therap* OR program* OR strateg*) AND (“lived experience*” OR “patient reported outcome*” OR “self reported outcome*” OR “quality of life” OR “mental wellbeing” OR satisfaction OR dissatisfaction OR “psychological impact*” OR “body image” OR “self image” OR ((relationship OR partner) NEAR satisfaction)))

**Medline (938)**

((“sexual well-being” OR “sexual wellbeing” OR “sexual health” OR “sexual satisfaction” OR “sexual function*” OR “sexual wellness”) AND (cancer N5 (genitourinary OR prostate OR renal OR bladder OR testicular OR penile)) AND (intervention* OR treatment* OR therap* OR program* OR strateg*) AND (“lived experience*” OR “patient reported outcome*” OR “self-reported outcome*” OR “quality of life” OR “mental wellbeing” OR satisfaction OR dissatisfaction OR “psychological impact*” OR “body image” OR “self-image” OR ((relationship OR partner) N5 satisfaction)))

**Scopus (729)**

TI:AB ((“sexual well-being” OR “sexual wellbeing” OR “sexual health” OR “sexual satisfaction” OR “sexual function*” OR “sexual wellness”) AND (cancer W/5 (genitourinary OR prostate OR renal OR bladder OR testicular OR penile)) AND (intervention* OR treatment* OR therap* OR program* OR strateg*) AND (“lived experience*” OR “patient reported outcome*” OR “self-reported outcome*” OR “quality of life” OR “mental wellbeing” OR satisfaction OR dissatisfaction OR “psychological impact*” OR “body image” OR “self-image” OR ((relationship OR partner) W/5 satisfaction)))
