# Supplementary material for: Experiences of sexual well-being interventions in males affected by genitourinary cancers and their partners: an integrative systematic review
Source: Support Care Cancer. 2023 Apr 14;31(5):265. doi: 10.1007/s00520-023-07712-8 (PMC10104925; doi:10.1007/s00520-023-07712-8)
Supplement: Supplementary file 2 — Supplementary file2 (DOCX 24 KB) [file 520_2023_7712_MOESM2_ESM.docx]

| **Supplementary table 2: Qualitative Findings** | | | | | |
| --- | --- | --- | --- | --- | --- |
| **Study** |  | **Evidence** | | | **Label** |
|  |  | **Unequivocal** | **Equivocal** | **Not supported** |  |
| **Study Bossio et al (2021)** | | | | | |
| **Finding** | **Communication with patient /partner** | | | | |
| Illustration | “We can’t even seem to have a conversation about (sexual intimacy). Like if I raise it, it goes nowhere…I sort of leave it up to him to raise it as an issue or a subject. And that’s not happening, so it’s basically just this -you know it’s just at the point where there’s just not a conversation” p8 | X |  |  | F1 |
| **Finding** | **Education/Support (Group format)** | | | | |
| Illustration | “Part of it is trusting the other couples are discreet. And you’re building a very strong relationship there sharing things, you don’t share sometimes with friends. So, there could be a bit of fear with a larger group, but it was comfortable “p 11 | X |  |  | F2 |
| **Finding** | **Timing of delivery (diagnosis)** | | | | |
| Illustration | “That’s life it’s the same as when you go into your operation. The most important thing is that you survive it. The second most important thing is that you don’t have any incontinence. And the third important thing is that you want to have sex.” p 8 | X |  |  | F3 |
| **Study** | **Letts et al (2010)** | | | | |
| **Finding** | **Communication with patient /partner** | | | | |
| Illustration | Patient Sexual changes “I would have to said that this hasn’t really affected her at all. But I haven’t discussed that with her either so I don’t know for sure” p500 | X |  |  | F6 |
| **Finding** | **Communication with/Physicians** |  |  |  |  |
| illustration | “Well, I kept asking the doctors is this going to affect? … Oh No. But the doctors never discuss this… and that is the way it was but nobody discusses it” Well, I could deal with it …. If somebody would have told you, eh? It was like dropping off, stepping off a cliff, and they say, well, you are going to fall down. But nobody said anything nobody said a word, it seems like well you’ll find out” p502 | X |  |  | F7 |
| **Finding** | **Education & Support** | | | | |
| Illustration | Effectiveness of treatment: I mean they get you believing that if you do these things it is going to work, but it doesn’t p501 | X |  |  | F8 |
| illustration | Lack of information: , “that this is to be expected” Doctor asked How is your sex life?  “Not great” he took this as an answer. He didn’t delve into it with any depth” p503 | X |  |  | F9 |
| illustration | Pre-treatment education - “This is not my job to make sure you are going to have sexual functioning. My job is to save your life”p502 |  | X |  | F10 |
| illustration | Post treatment I told him the problems I was having with changes in my sex life and he said “well I had another patient that went through that and he let it ruin his life. “He said don’t let that happen to you and that was his friendly advice to me. And the topic changed after that. p503 | X |  |  | F11 |
| **Study** | **Mehta et al (2019)** | | | | |
| **Finding** | **Communication with patient /partner** | | | | |
| Illustration | He just doesn’t just have no desire to have sex, he has no desire to hug me, kiss me, cuddle me. That’s what breaks my heart …. that has always meant more to me than actual “p 188 | X |  |  | F12 |
| **Finding** | **Communication with Physician** | | | | |
| Illustration | “My doctor was great about a lot of information but that particular piece (around sexual side effects) was not really addressed p187 | X |  |  | F13 |
| illustration | Gay men & same sex Couples: “Talking about having an orgasm, and not having any fluid being important for gay men vs um straight couple” p188 | X |  |  | F14 |
| **Finding** | **Education & Support** |  |  |  |  |
| illustration | “You feel comfortable when you know somebody else is going through the same thing that you are going through. I think that helps” p 188 | X |  |  | F15 |
| **Findings:** | **Timing/Delivery** |  |  |  |  |
| illustrations | “I think if there was something that we would have liked differently with the whole procedure and recovery process is to have a website that has all of this stuff… that has people to talk to, that has a video instruction, that has imagery… outlets for emotional support, like that kind of stuff would have really helped” p188 |  | X |  | F16 |
| **Study:** | **O’Brien et al (2011)** | | | | |
| **Finding** | **Communication with patient /partner** | | | | |
| ilustration | I don’t know whether the team (at the hospital) took the attitude he’s getting on for 80. It’s not worthwhile bothering much with him”. They didn’t ask me I had to tell them that I couldn’t function sexually at all” p 203 | X |  |  | F17 |
| **Finding** | **Communication with health care professional** | | | | |
| illustration | “I feel that they could do with opening up the discussion but they won’t discuss it… You can mention it and they shrug their shoulders, some of them, and say “it doesn’t bother me anymore” a large number of men will say that. Therefore, one feels that’s the end of discission” p203 |  | X |  | F18 |
| **Finding** | **Education & Support** |  |  |  |  |
| Illustration | Lack of continuity of care:” I think if I saw the same person each time you would probably build a rapport with that person. But seeing a stranger every time you’re thinking oh my god. I’m not going to walk in there and start talking about my sex life with somebody I’ve never seen in my life before” … I think I glossed over it (the psychological impact) | X |  |  | F19 |
| **Finding** | **Timing /Delivery** | | | | |
| Illustrations | “You simply don’t know how you will react to it yourself…I think it’s only later on that I was affected emotionally by it” p 203 | X |  |  | F20 |
| **Study Wittman et al (2015) a** | | | | | |
| **Finding** | **Communication with patient & partner** | | | | |
| Illustration | “I think I have some feelings of grief in regard to the sexual aspect because I think we have a good sexual life; I think that it’s an important part of our relationship p 498 | X |  |  | F21 |
| **Finding** | **Education & Support- high expectation of erectile function Pre op** | | | | |
| illustration | “Yeah, as of now, I’m still of the optimistic thinking that were not going to need that (sexual aides)” p498 | X |  |  | F22 |
| **Finding** | **Education & Support- sexual function post-op** | | | | |
| illustration | “Well, that seems like another apparatus that it’s like so much work, you know” p498 | X |  |  | F23 |
| **Study Wittman et al 2015 (b)** | | | | | |
| **Finding** | **Communication patient /partner- pre-Operative barriers** | | | | |
| illustration | “Uh there were lots of times …that I would have preferred to have sex but it just not worth the hassle” p 163 | X |  |  | F24 |
| **Finding** | **Timing /Delivery Pre-Operative barriers** | | | | |
| illustration | “My problem is during sex, I lose my erection because I am afraid if I don’t ejaculate soon, I’m going to hurt her… the next thing I know, I’ve lost my erection p163 | X |  |  | F25 |
